# Decolorization of Solophenyl Red 3BL Polyazo Dye by Laccase-Mediator System: Optimization through Response Surface Methodology

**DOI:** 10.4061/2011/179050

**Published:** 2011-08-02

**Authors:** Mohamed Neifar, Atef Jaouani, Amel Kamoun, Raoudha Ellouze-Ghorbel, Semia Ellouze-Chaabouni

**Affiliations:** ^1^Unité Enzymes et Bioconversion, Ecole Nationale d'Ingénieurs de Sfax, Université de Sfax, Route de Soukra, Sfax 3038, Tunisia; ^2^Laboratoire Microorganismes et Biomolécules Actives, Faculté des Sciences de Tunis, Université de Tunis, Campus Universitaire, Tunis 2092, Tunisia; ^3^Laboratoire de Chimie Industrielle, Ecole Nationale d'Ingénieurs de Sfax, Université de Sfax, Route de Soukra, Sfax 3038, Tunisia

## Abstract

The decolorization of direct Solophenyl red 3BL (SR), a polyazo dye extensively used in textile industry was studied. The *Fomes fomentarius* laccase alone did not decolorize SR. The natural redox mediator, acetosyringone (AS), was necessary for decolorization to occur. Box-Behnken design was used to evaluate the effects of three parameters, namely, enzyme concentration (0.5–2.5 U mL^−1^), redox mediator concentration (3–30 *μ*M), and incubation time (1–24 h), on the SR decolorization yield. The fitted mathematical model allowed us to plot response surfaces as well as isoresponse curves and to determine optimal decolorization conditions. The results clearly indicated that the AS concentration was the main factor influencing the SR decolorization yield. The selected optimal conditions were enzyme concentration 0.8 U mL^−1^, mediator concentration 33 *μ*M, and time 14 h 30 min. These conditions allowed 79.66% of SR decolorization versus 80.70% for the predicted value. These results showed a promising future of applying laccase-AS system for industrial wastewater bioremediation.

## 1. Introduction

Waste waters of the textile industries, well implanted in Tunisia, contain considerable amounts of nonfixed dyes and especially of azo dyes. The release of those colored waste waters in the ecosystem is a dramatic source of esthetic pollution, eutrophication, and perturbations in the aquatic life [1]. 

Most physicochemical dye removal methods, which are generally used for the effluent treatment, have many limitations [2, 3]. Biological decolorization and degradation is an environmental-friendly and cost-competitive alternative to the physicochemical decomposition process [4].

The dye biodegradation is carried out mostly by white rot fungi and by their ligninolytic enzymes such as lignin peroxidases, manganese peroxidases, and laccases [5, 6]. Laccases are oxidoreductases that belong to the multinuclear copper-containing oxidases and are able to decolorize and detoxify industrial dyes [7, 8]. However, some of the dyes cannot be oxidized, or partly oxidized by laccase because they are too large to penetrate into the enzyme active site or have a particularly high redox potential. Laccase mediators, such as 1-hydroxybenzotriazole (HBT), 2,2-azino-bis 3-ethylbenzothiaoline-6-sufonic acid (ABTS), and Remazol Brilliant Blue R (RBBR), are found to extend or permit the oxidation of nonspecific substrate by laccase [8, 9].

Nevertheless, laccase-mediator system has not yet been applied at large scale due to the cost of mediators and their toxicity [10]. In recent years, some natural phenolic compounds, including syringaldehyde and acetosyringone (AS), have been described as efficient and ecofriendly laccase mediators for textile and environmental applications [11].

Previous studies have shown that the efficiency of the dye decolorization by laccase depends on many factors such as the reaction time, the concentration of the enzyme and the structure and the concentration of the dye, and the redox mediator [12, 13]. Response surface methodology (RSM) is an efficient experimental strategy to determine optimal conditions for a multivariable system rather than optimizing by the conventional method which involves changing one independent variable while keeping the other factors constant. These conventional methods are time consuming and incapable of detecting the true optimum [14–17]. The RSM has been successfully applied in optimization of the experimental conditions of the dye decolorization with fungal laccases [12, 13].

The white-rot fungus *Fomes fomentarius* has been recently described as a good producer of laccase in solid-state cultures [18, 19]. It has also been reported that *F. fomentarius* laccase efficiently decolorizes the anthraquinone dye Remazol Brilliant Blue R without mediators [20]. The present work focused on applying laccase from *F. fomentarius* combined with the natural mediator AS, to decolorize C.I. Direct Solophenyl red 3BL polyazo dye. The main objectives of this work were to better understand the relationships between the decolorization variables (enzyme concentration, redox mediator concentration, and incubation time) and the response (SR decolorization yield) and to obtain the optimum experimental conditions for decolorization using a three-level Box-Behnken design and the RSM. All the results obtained in this study would provide a sound basis for further exploration.

## 2. Experimental Section

### 2.1. Chemicals

**Figure 1:**
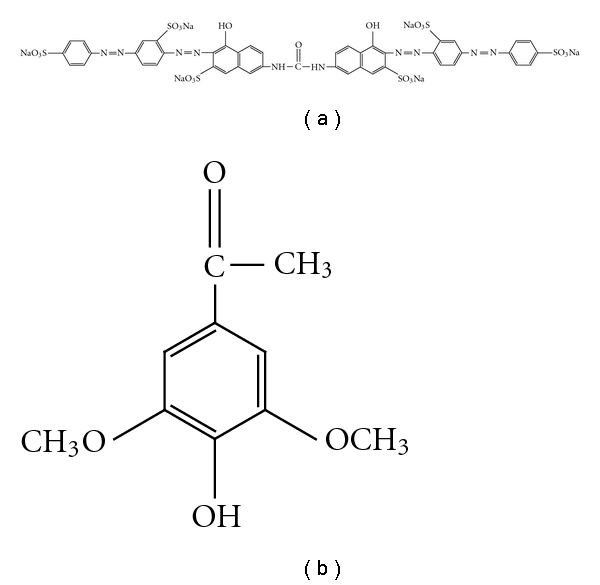
Chemical structures of (a) the polyazo dye Solophenyl red 3BL (C.I. Direct 80) and (b) the natural redox mediator acetosyringone.

Solophenyl red 3BL (C.I. Direct 80) tetraazo dye (Figure 1(a)) was obtained from the Ciba-Geigy and used without further purification. This dye was chosen as a model compound of polyazo dyes. Acetosyringone (Figure 1(b)), purchased from Sigma-Aldrich was assayed as a natural mediator for solophenyl red decolorization. All the other reagents used were of highest purity grade available commercially. 

### 2.2. Enzyme Preparation

*F. fomentarius* laccase was produced on wheat bran solid medium, and the crude extract was fractioned by ammonium sulfate precipitation as previously described [18]. Laccase activity was assayed using 5 mM 2, 6-dimethoxyphenol (DMP) in 100 mM sodium tartrate buffer, pH 4.5 (*ε*_469_ = 27,500 M^−1^ cm^−1^, referred to DMP concentration). The enzymatic reactions were carried out at room temperature (22–25°C) and one unit of enzyme activity was defined as the amount of enzyme oxidizing 1 *μ*mol of substrate per minute [21].

### 2.3. Dye Decolorization Test

The reaction mixture for SR decolorization experiments contained 100 mM tartrate buffer (pH 4.5), laccase (0.5–2.5 U mL^−1^), and AS (3–30 *μ*M). SR concentration was selected in order to obtain 1.4 absorbance units at the dye maximum absorbance wavelength, 543 nm (0.14 g L^−1^, final concentration). All the reactions were incubated at 30°C in complete darkness and the residual dye concentration was determined at different incubation times (1–24 h) by monitoring the decrease in absorbance at 543 nm using a Shimadzu UV-VIS Scanning spectrophotometer (UV-2101-PC). Dye decolorization was expressed in terms of percentage. A control test containing the same amount of a heat-denatured laccase was performed in parallel, and, in order to find the effect of AS, experiments were also conducted without addition of AS.

### 2.4. Experimental Design and Statistical Analysis

In this work, a Box-Behnken design [15–17, 22–24] was set up to look for the best experimental conditions of three independent factors affecting the efficiency of the decolorization of SR, namely: enzyme concentration (U_1_), redox mediator concentration (U_2_), and incubation time (U_3_) (Table 1). The relationship between the response (SR decolorization yield) and the three quantitative variables was approximated by the following second-order polynomial function:

(1) $$\begin{array}{cc} \eta = & \beta_{0}+\beta_{1}{\text{X}}_{1}+\beta_{2}{\text{X}}_{2}+\beta_{3}{\text{X}}_{3}+\beta_{11}{\text{X}}_{1}^{2}+\beta_{22}{\text{X}}_{2}^{2}+\beta_{33}{\text{X}}_{3}^{2}\\ & +\beta_{12}{\text{X}}_{1}{\text{X}}_{2}+\beta_{13}{\text{X}}_{1}{\text{X}}_{3}+\beta_{23}{\text{X}}_{2}{\text{X}}_{3}, \end{array}$$

where *η* represents the theoretical response;  *β*_0_, *β*_*j*_,  *β*_*jk*_, and *β*_*jj*_ are model coefficients. *X*_*j*_ are coded variables related to the natural variables *U*_*j*_  by the following equation:

(2) $$\;X_{j}=\frac{Uj-\text{Center}\left(j\right)}{\text{Step}\;\text{of}\;\text{variation}\left(j\right)},$$

where Center (*j*) = (*U*_*j*,high_ − *U*_*j*,  low_)/2, Step of variation (*j*) = (*U*_*j*,high_ + *U*_*j*,  low_)/2  *U*_*j*,high_ and *U*_*j*,low_: two extreme levels (high and low) given for each natural variable *U*_*j*_. 

The coded variables *X*_*j*_ are equal to −1 and +1 when the levels of natural variable *U*_*j*_ are *U*_*j*  low_ and *U*_*j*  high_, respectively.

The observed response *y*_*i*_ for the *i*th experiment is

(3) $$y_{i}=\eta_{i}+e_{i}\;\left(e_{i}\;\text{is}\;\text{experimental}\;\text{error}\right).$$

From the experimental results (*y*), the estimates (*b*_0_, *b*_1_, *b*_2_, …) of the model coefficients are calculated and the model can be written as follows:

(4) $$\begin{array}{cc} \hat{y} & =b_{0}+b_{1}X_{1}+b_{2}X_{2}+b_{3}X_{3}+b_{11}X_{1}^{2}+b_{22}X_{2}^{2}+b_{33}X_{3}^{2}\\ & \;+b_{12}X_{1}X_{2}+b_{13}X_{1}X_{3}+b_{23}X_{2}X_{3}, \end{array}$$

where,$\;\hat{y}$ is the estimated response function; *b*_0_, *b*_*j*_, *b*_*jk*_, and *b*_*jj*_ are the estimated model coefficients.

A three-variable Box-Behnken design with 17 experiments (Table 2) was used to estimate the model coefficients. The experimental points are located in the middle of a cube ridges (12 experiments: runs no. 1 to 12) and at the center of the cube (5 experiments: runs no. 13 to 17). The five replicates at the center point were carried out in order to estimate the pure error variance [16, 17, 24]. 

The significance of the fitted model was tested by the mean of the analysis of variance (ANOVA) [16, 17, 24]. The model adequacy was checked, before a predictive use of it in the studied domain, using four test points (runs n° 18 to 21) [17].

The fitted model was used to study the relative sensitivity of the response to the variables in the whole domain and to look for the optimal experimental conditions. The relationship between the response and the experimental variables was illustrated graphically by plotting the isoresponse curves and the response surfaces [25, 26].

In this study, the generation and the data treatment of the Box-Behnken design were performed using the experimental design software NemrodW [27].

## 3. Results and Discussion

### 3.1. Preliminary Study

Some fungal laccases as well as laccase mediator systems are efficient in dye decolorization. Different dyes were decolorized by different laccases at different rates. The decolorization rate depends on the structure and the redox potential of the enzyme as well as the dye structure [28–30]. The polyazo dye SR is widely used for textile dyeing process which is biodegradation resistant. Preliminary results showed that *F. fomentarius* laccase did not decolorize SR (data not shown), indicating that the presence of a mediator is required. Similarly, reports from the literature show that laccase alone does not decolorize some types of textile dyes [9, 12]. The reason may be that the redox potential of the dye is higher than that of type 1 Cu of the laccase or the dye could not access the type 1 Cu active site because of its steric hindrance. However, such dyes can be oxidized by laccase in the presence of some redox mediators [9–12]. In the present study, the effect of ABTS, RBBR, and AS on SR decolorization was assessed at a concentration of 10 *μ*M. Among the three different redox mediators tested only AS showed the highest decolorization yield after 1h of incubation (29% for AS versus 21 and 16% for ABTS and RBBR, resp.). Thus further experiments for the experimental design were carried out with the natural mediator acetosyringone.

### 3.2. Estimated Model

Twenty-one experiments were carried out. The experimental conditions, shown in Table 2, were arranged according to the three-variable Box-Behnken design. The corresponding observed values of the decolorization yield are indicated in the last column of Table 2. The observed responses were used to compute the model coefficients using the least square method. This allowed us to write the following estimated model:

(5) $$\;\begin{array}{cc} \hat{y} & =55.748-2.836X_{1}+23.300X_{2}\\ & \;+\;7.584X_{3}-2.283X_{1}^{2}\;-6.690X_{2}^{2}\\ & \;-9.853X_{3}^{2}-4.520X_{1}X_{2}-2.582X_{1}X_{3}\\ & \;+1.155X_{2}X_{3}. \end{array}\;\;$$

### 3.3. Statistical Analysis and Validation of the Model

The analysis of variance for the fitted model (Table 3) showed that the regression sum of squares was statistically significant at the level 99.9% and the lack of fit was not significant. Thus, we concluded that the model represented well the measured data.

In addition, numerical results for check points (Table 4) showed that the measured values were very close to those calculated using the model equation. Indeed, the differences between calculated and measured responses were not statistically significant when using the *t*-test at a 95% probability level, as shown in Table 4. Therefore, the estimated model coefficients could be recalculated with all the 21 experiment results. The corresponding second-order model is represented by the following equation:

(6) $$\begin{array}{cc} \hat{y} & =\;56.305-2.872X_{1}+23.227X_{2}\\ & \;+\;7.508X_{3}-2.454X_{1}^{2}-6.908X_{2}^{2}\;\\ & \;-\;10.203X_{3}^{2}-4.502X_{1}X_{2}-2.570X_{1}X_{3}\\ & \;+\;1.180X_{2}X_{3}. \end{array}\;\;$$

### 3.4. Interpretation of the Response Surface Model

**Figure 2:**
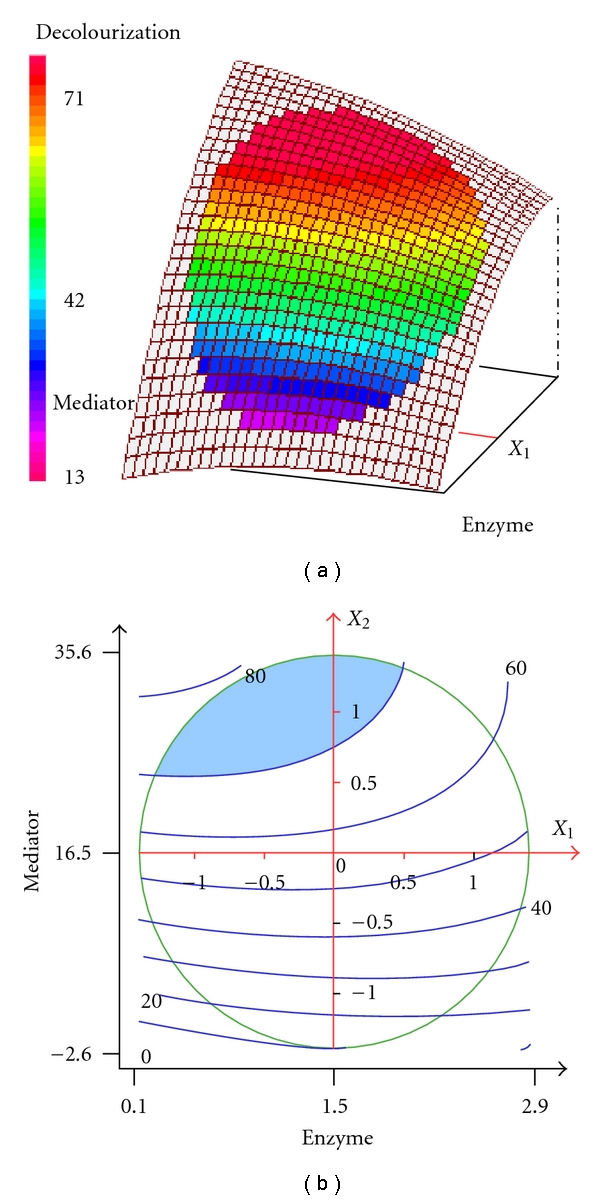
Three-dimensional response surface and contour plots for the effect of enzyme and redox mediator concentrations at constant incubation time (12.5 h) on the decolorization of SR.

**Figure 3:**
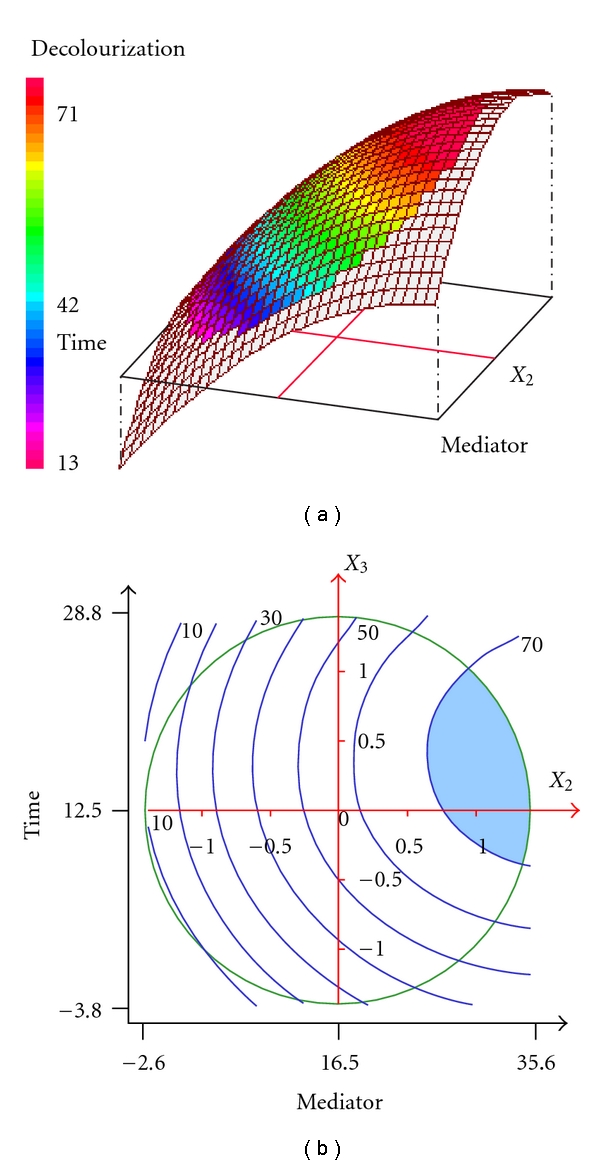
Three-dimensional response surface and contour plots for the effect of redox mediator concentration and incubation time at constant enzyme concentration (1.5 U/mL) on the decolorization of SR.

**Figure 4:**
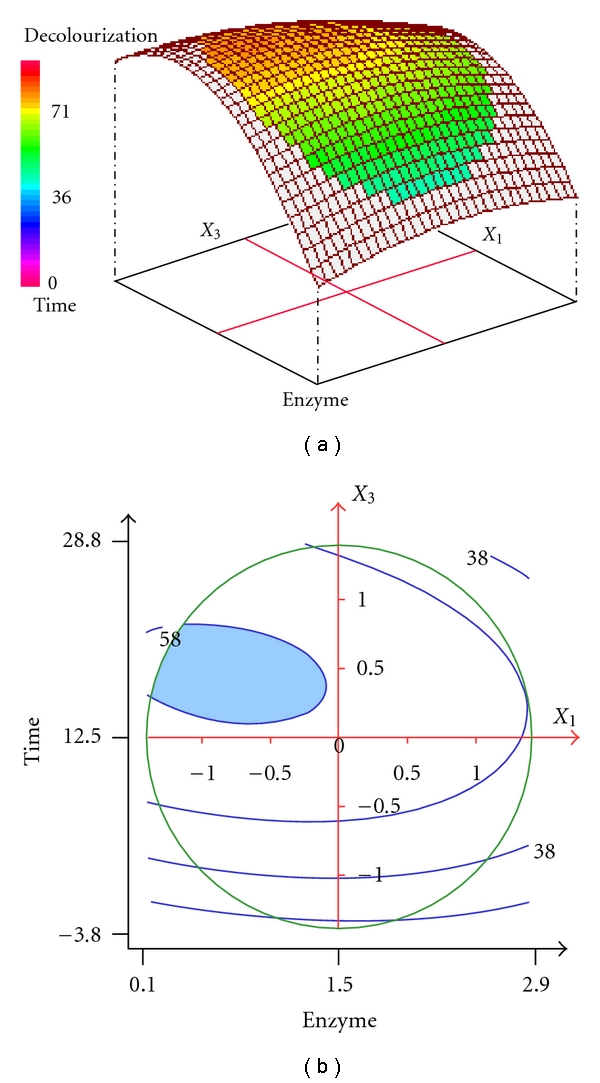
Three-dimensional response surface and contour plots for the effect of enzyme concentration and incubation time at constant redox mediator concentration (16.5 *μ*M) on the decolorization of SR.

The relationship between the response and the experimental variables can be illustrated graphically by plotting three-dimensional response surface plots and the two-dimensional isoresponse curves (Figures 2 to 4). In these plots, the factor not represented by the two axes was fixed at its 0 coded level. Such plots are helpful in studying the effects of the variation of the factors in the domain studied and consequently, in determining the optimal experimental conditions [25, 26]. 

Figure 2 shows the effect of enzyme concentration (*X*_1_) and mediator concentration (*X*_2_) on SR decolorization yield at 12 h 30 minutes (*X*_3_ = 0). It clearly shows that the decolorization yield increases with the mediator concentration. However, in the presence of a large amount of mediator (>16.5 *μ*M), the enzyme concentration exhibits a negative effect on the decolorization yield. At 12 h 30 minutes, the decolorization yield can reach 70 to 80% (blue coloured area) when we use a mediator concentration in the range of 20–30 *μ*M and enzyme concentration lower than 2 U/mL. Wong and Yu [31] also reported that the efficiency of laccase-mediator systems in the decolorization reaction depended principally on the mediator concentrations and laccase activity used. The feasibility of the laccase-mediator systems in biotransformation reactions depends on redox reversibility of the radical-substrate reaction, as well as on the balance between the stability and reactivity of the mediator radical which, in addition, should not inhibit enzyme activity [32]. 

The positive effect of mediator concentration was also demonstrated in Figure 3. When the enzyme concentration was fixed at 1.5 U/mL (*X*_1_ = 0), the increase of mediator concentration and incubation time improved the SR decolorization yield. Experimentations conducted with a mediator concentration more than 20 *μ*M and incubation time in the range of 10–25 h led to relatively high decolorization yields (70–77%) as shown in Figure 3 (blue coloured area). 

Figure 4 represents the effect of enzyme concentration (*X*_1_) and incubation time (*X*_3_) on SR decolorization at constant redox mediator concentration (16.5 *μ*M). The contour plots of Figure 4 also support the important role of incubation time. Indeed, the decolorization yield increases from 28 to 58% when the incubation time increases from 1 h to 13 h 30 minutes. High decolorization yields 58–60% (blue coloured area) can be reached when using a relatively low enzyme concentration (<1.5 U/mL) and an incubation time in the range of 13 h 30 minutes–22 h. 

The results presented above showed that the concentration of the AS phenolic mediator was the more relevant factor for the SR decolorization. According to the relevant literature, the action mechanism of phenolic mediators should be similar to that of –N (OH)– type mediators (like HBT) [33]. After its oxidation by laccase to cation radical, the small AS molecule, can transfer electrons between the enzyme and the dye as a redox mediator and thus oxidizes the nonsubstrate dye. The fact that *F. fomentarius* laccase is able to decolorize the SR polyazo dye with AS as a mediator makes it very interesting, since this phenolic mediator can be easily obtained from natural substrates by organic extraction or alkaline treatment. Also, the use of this natural mediator may be a solution to the toxicity problem of the synthetic mediators currently used for the textile effluent treatment [8, 32].

### 3.5. Optimization

The selection of optimal conditions was based on the determination of the experimental conditions leading simultaneously to the maximization of the SR decolorization and the minimization of the process cost. 

**Figure 5:**
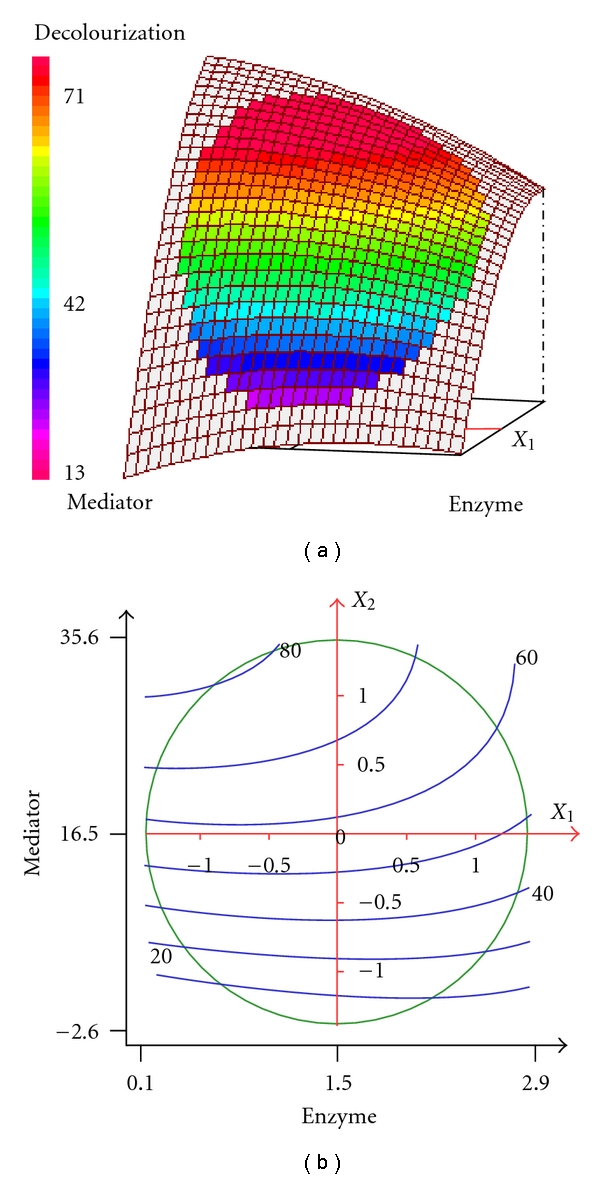
Three-dimensional response surface and contour plots for the effect of enzyme and redox mediator concentrations at constant incubation time (14.5 h) on the decolorization of SR.

As the SR decolorization yield can be maximized when using an incubation time in the range of 10 h–25 h (Figure 3) and 13 h 30 minutes–22 h (Figure 4), we fixed the incubation time at a relatively low level (14 h 30 minutes) in order to lower the process cost, and we ploted enzyme versus redox mediator concentration (Figure 5) to look for the highest decolorization yield. 

Figure 5 shows that the optimal conditions are enzyme concentration 0.8 U mL^−1^, mediator concentration 33 *μ*M, and reaction time 14 h 30 minutes. Under these conditions, the expected value of the SR decolorization yield was$\;{\hat{y}}_{\text{op}}=80.70\%\pm 0.75$. A supplementary experiment was carried out under the selected optimal conditions. It led to an experimental SR decolorization yield equal to 79.66%, which was in close agreement with the predicted value. A similar decolorization yield (81.12 %) was obtained when using the purified laccase from *F. fomentarius* [20] under the optimal conditions.

## 4. Conclusion

This work revealed that the response surface methodology was a useful tool to determine the optimal experimental conditions for the decolorization of the commercially available textile polyazo dye, solophenyl red (SR). The presence of a natural mediator, acetosyringone (AS), was essential for the decolorization of RB-5 by *F. fomentarius* laccase. The concentration of the AS proved to be the principal factor that affected the yield of the dye decolorization. The selected optimal conditions (enzyme concentration 0.8 U mL^−1^, mediator concentration 33 *μ*M, and time 14 h 30 minutes) were checked and confirmed by supplementary experiments using partially and purified *F. fomentarius* laccases. The experimental response value obtained with partially purified laccase (79.66%) was found to be in good agreement with the predicted one (80.70%). Similar decolorization yield (81.12%) was obtained when using the purified laccase from *F. fomentarius* under the optimal conditions. 

From a standpoint of a real case application, the results showed that laccase-AS system proved to be efficient for solutions of dyes currently used in textile industries. Further pilot scale studies are required with this biocatalytic process for actual industrial applications, and detailed study is needed to explore the mechanism involved.

## Figures and Tables

**Table 1:** Experimental domain of the Box-Behnken design.

Variable	Factor	Unit	Center	Step of variation
*X*_1_	Enzyme conc.	U/mL	1.5	1.0
*X*_2_	Mediator conc.	*μ*M	16.5	13.5
*X*_3_	Time	h	12.5	11.5

**Table 2:** Experimental conditions of the Box-Behnken design in coded and natural variables and the corresponding experimental and theoretical responses.

	Run N°	*X*_1_	*X*_2_	*X*_3_	Enzyme	Mediator	Time	Measured decolorization	Estimated decolorization
					(U/mL)	(*μ*M)	(h)	(%)	(%)
	1	−1.00	−1.00	0.00	0.5	3.0	12.5	18.36	21.79
	2	1.00	−1.00	0.00	2.5	3.0	12.5	26.42	25.16
	3	−1.00	1.00	0.00	0.5	30.0	12.5	76.17	77.43
	4	1.00	1.00	0.00	2.5	30.0	12.5	66.15	62.72
	5	−1.00	0.00	−1.00	0.5	16.5	1.0	36.72	36.28
	6	1.00	0.00	−1.00	2.5	16.5	1.0	31.52	35.78
	7	−1.00	0.00	1.00	0.5	16.5	24.0	60.87	56.62
	8	1.00	0.00	1.00	2.5	16.5	24.0	45.34	45.78
	9	0.00	−1.00	−1.00	1.5	3.0	1.0	12.47	9.48
	10	0.00	1.00	−1.00	1.5	30.0	1.0	54.59	53.77
	11	0.00	−1.00	1.00	1.5	3.0	24.0	21.51	22.33
	12	0.00	1.00	1.00	1.5	30.0	24.0	68.25	71.24

Center points	13	0.00	0.00	0.00	1.5	16.5	12.5	60.44	55.75
	14	0.00	0.00	0.00	1.5	16.5	12.5	55.71	55.75
	15	0.00	0.00	0.00	1.5	16.5	12.5	51.59	55.75
	16	0.00	0.00	0.00	1.5	16.5	12.5	53.68	55.75
	17	0.00	0.00	0.00	1.5	16.5	12.5	57.32	55.75

Check points	18	−0.40	−0.25	−0.17	1.1	13.1	10.5	51.59	48.12
	19	0.40	−0.25	−0.17	1.9	13.1	10.5	49.82	47.12
	20	0.00	0.45	−0.17	1.5	22.6	10.5	65.24	63.22
	21	0.00	0.00	−0.17	1.5	16.5	10.5	51.65	54.17

**Table 3:** Analysis of variance.

Source of variation	Sum of squares	Degrees of freedom	Mean square	Ratio	Significance
Regression	5652,89	9	628,099	34.1739	***
Residuals	128,657	7	18,3795		
Validity	82,6035	3	27,5345	2.3915	N.S.
Error	46,0531	4	11,5132		

Total	5781,55	16			

***Significant at the level 99.9% N.S.: nonsignificant.

**Table 4:** Validation of the model with the check points.

Run N°	*y*_*i*_	${\hat{y}}_{i}$	$d=\left(y_{i}-\;{\hat{y}}_{i}\right)$	*t* exp.	Significance
18	51.590	48.122	3.468	0.741	N.S.
19	49.820	47.108	2.712	0.579	N.S.
20	65.240	63.216	2.024	0.432	N.S.
21	51.650	54.174	−2.524	−0.538	N.S.

*y*_*i*_: measured response value; ${\hat{y}}_{i}$: estimated response value; *d*: difference between measured and estimated response values; *t* exp.: student experimental value; N.S.: non significant.
